# Supplementary material for: Dissection of amino acid acquisition pathways demonstrates that amino acid starvation of Borrelia burgdorferi results in a (p)ppGpp-independent maladaptive response
Source: Commun Biol. 2025 Dec 23;9:105. doi: 10.1038/s42003-025-09374-0 (PMC12830385; doi:10.1038/s42003-025-09374-0)
Supplement: Supplementary file 2 — Supplementary Information [file 42003_2025_9374_MOESM2_ESM.pdf]

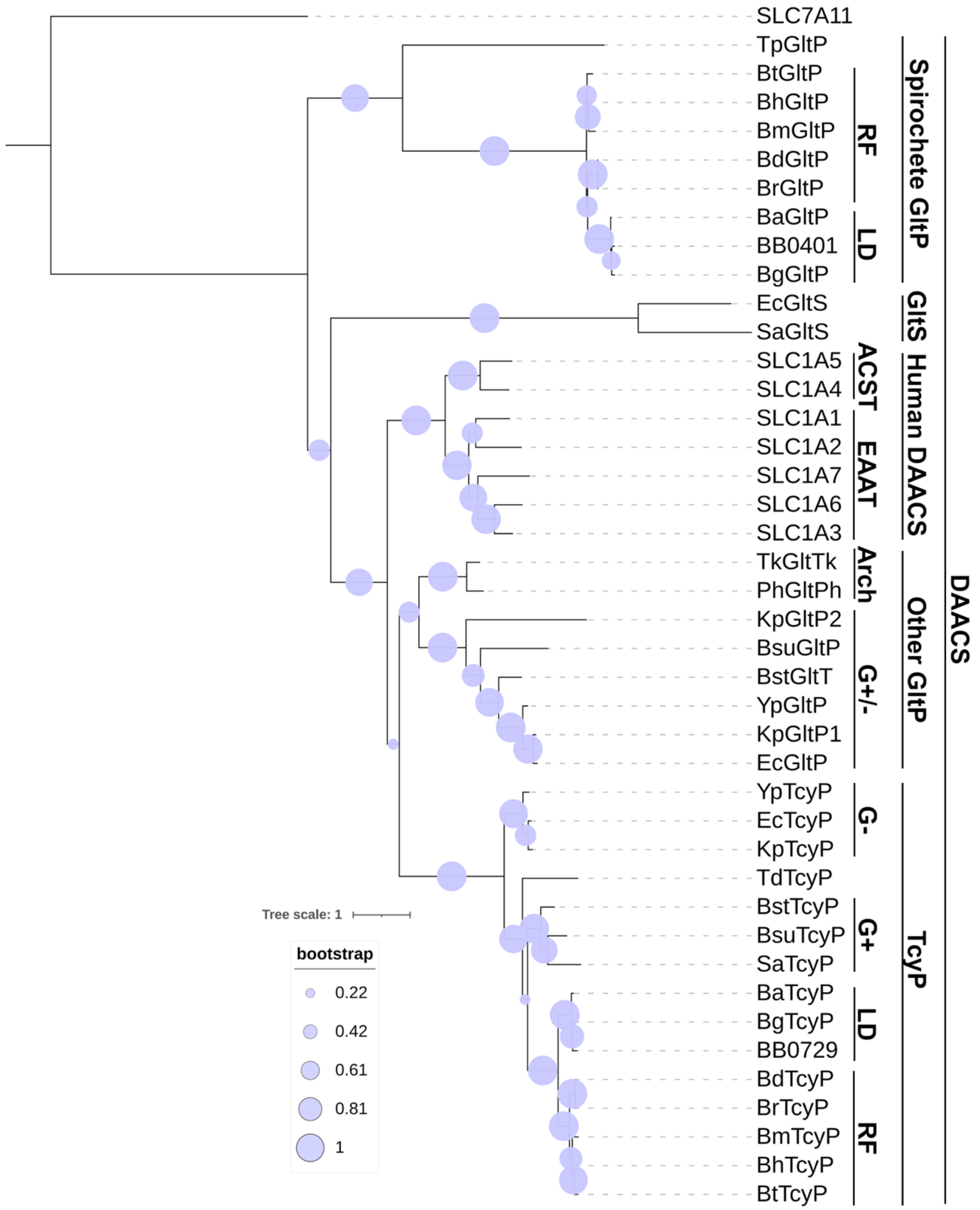

Fig S1: Phylogenetics of GltP and TcyP DAACs. a) Rooted phylogenetic tree of bacterial GltP, GltS, TcyP transporters. SLC7A11, a human cationic amino acid transporter (CAT) was used as an outgroup. Bootstrap values are represented as lilac spheres. Protein list with UniProt IDs can be found in Table S3.

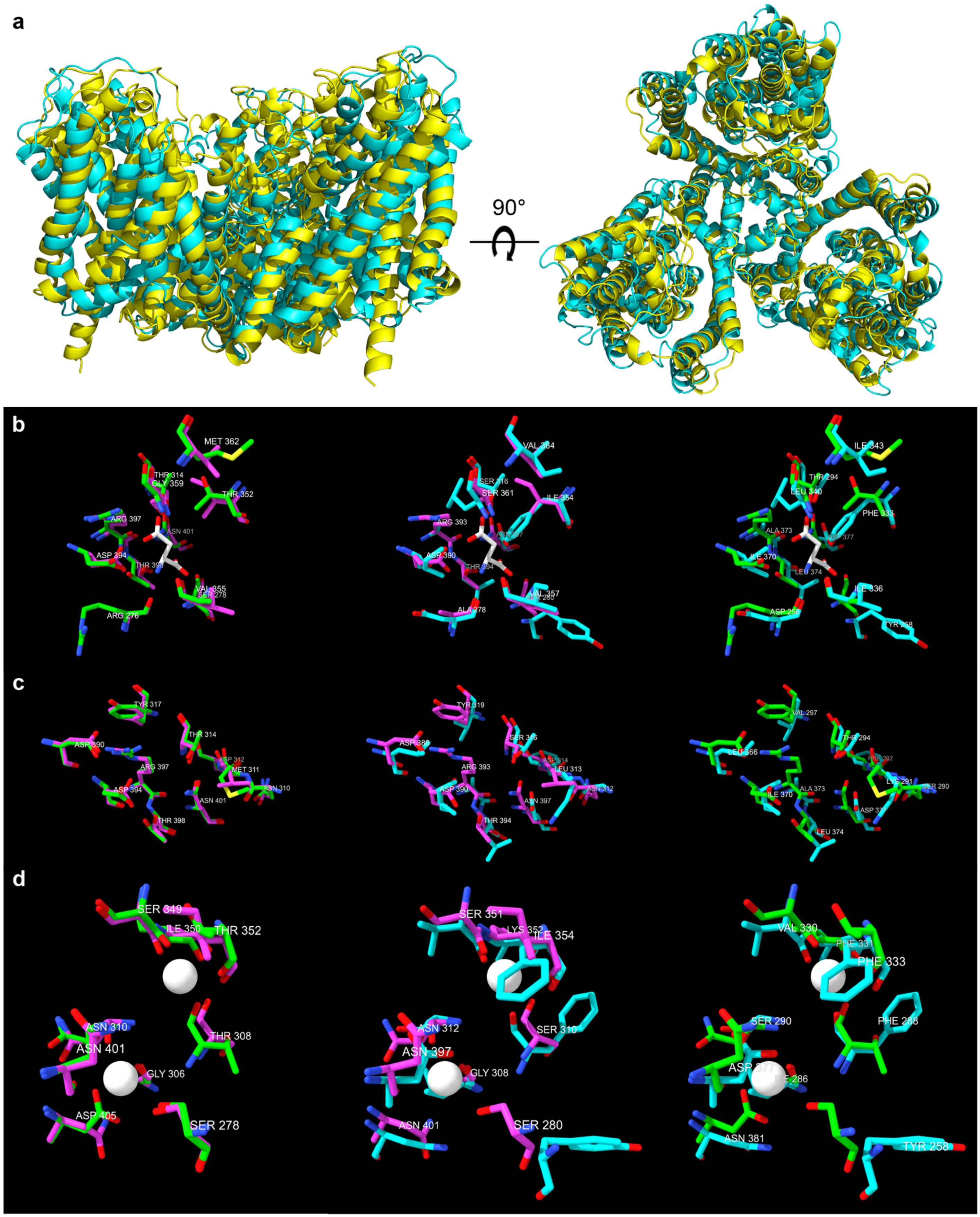

Fig S2: GltP models and binding sites. a) Overlay of BB0401 modeled against 2nwl[1] (cyan) and BB0401 as modeled by AlphaFold2-Multimer (yellow). b-d) Residue alignments for b) aspartate binding site complexed with Asp (white) from the 2nwl crystal structure, c) predicted glutamate binding site as predicted by Rahman et al.[2], and d) sodium binding sites 1 and 2 complexed with Na (white) from the 2nwx[1] crystal structure. Glt_{Ph} (2nwl or 2nwx)[1] is shown in green, $GltP_{Ec}$ model in magenta, and BB0401 model in cyan. Residues for GltPh are labeled in the first column, residues for GltPEc are labeled in the second column, and residues for BB0401 are labeled in the third column.†

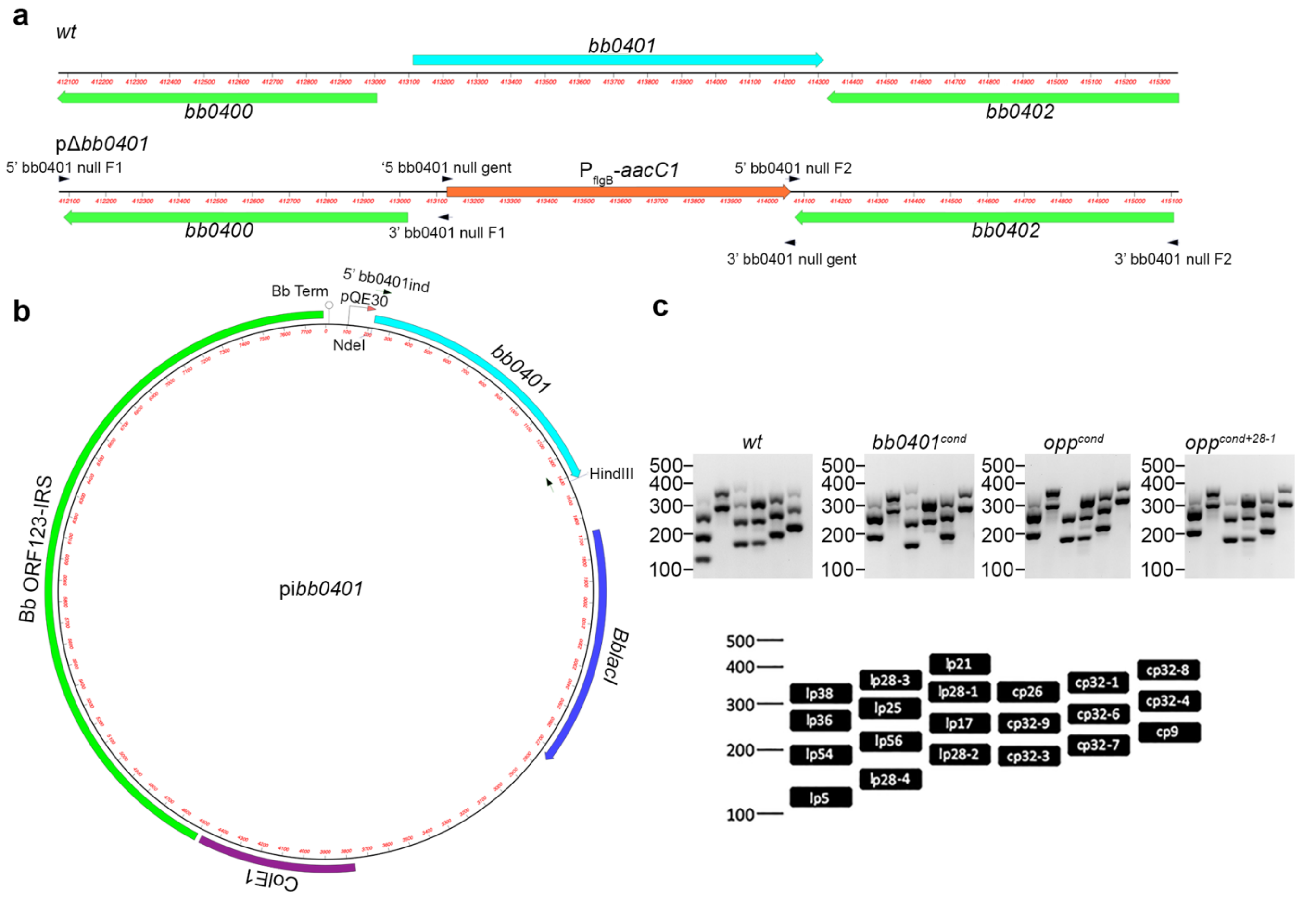

Fig S3: *bb0401* is essential for growth. a Schematic of *bb0401* locus on *B. burgdorferi* chromosome (*wt*) where gene of interest is in cyan and construction of pΔ*bb0401* where antibiotic cassette is in orange and arrows represent primer locations **b** Schematic of pi*bb0401* where gene of interest is in cyan, *lacI* in blue, *E. coli* origin of replication in purple, and cp9 shuttle vector region in green, arrows represent primer location, and restriction enzyme sites are shown. **c** Plasmid content multiplex PCRs for all strains, ladder sizes are shown in bp and a schematic of multiplex targets is displayed below.

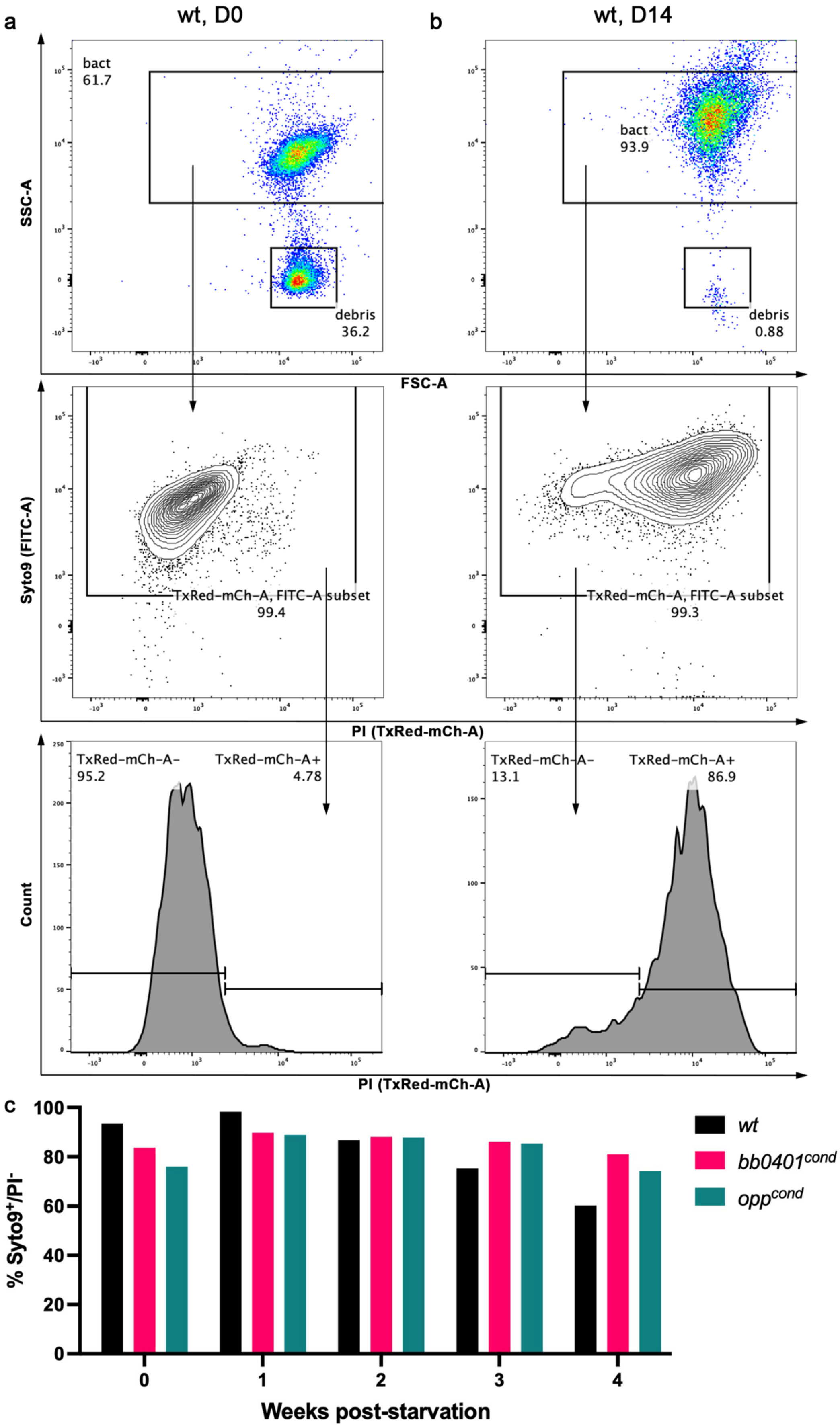

Figure S4: Amino acid transport is not essential for room temperature growth. a-b) Example gating strategy to identify live/dead cell populations using flow cytometry for *wt*. Top graph shows FSC-A/SSC-A for exclusion of debris present in the media, middle graph shows TxRed-mCh-A/FITC-A to identify the FITC-A+ subset. Bottom graph shows a histogram of TxRed-mCh-A to gate live and dead populations. c) Percent of live cells (Syto9+/PI-) during room temperature incubation sampled weekly over a four-week period. Two-way ANOVA found no statistical significance in pairwise comparisons.

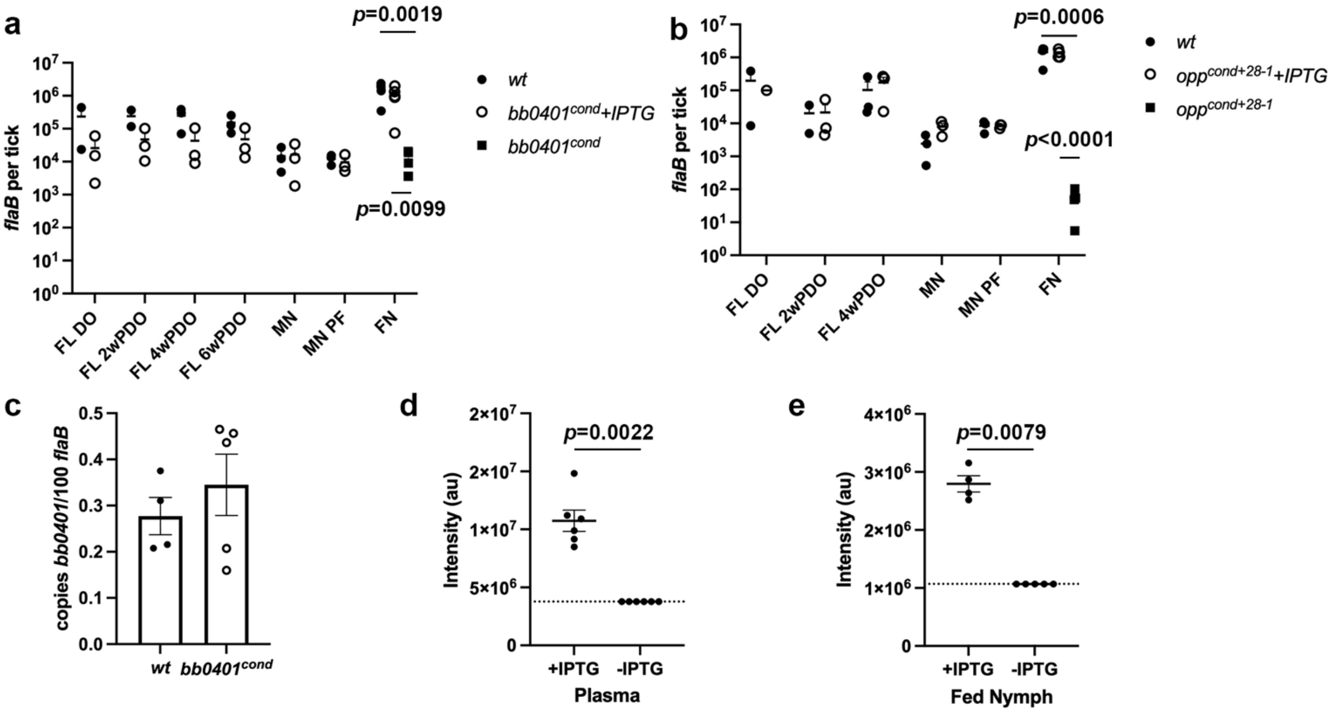

Figure S5: DNA burdens are lower in fed nymph for both mutants. Burdens as determined by qPCR of fed larvae at drop-off (FL DO), fed larvae at timepoints post-drop-off (FL #wPDO), post-molt nymphs (MN), molted nymphs prior to feeding (MN PF), and fed nymphs (FN) for a) *bb0401*^{comp} and b) *opp*$^{cond+28\text{-}1}$. c) *bb0401* transcripts in mouse hearts infected with *wt* or *bb0401*^{cond} +IPTG as measured by qRT-PCR and reported as copies of *bb0401*/*100 flaB*. IPTG detection in d) pooled mouse plasma and e) pooled fed nymphs. Dotted lined represent the LOQ. *p*-values were determined for pairwise comparisons using a two-tailed unpaired *t* test.

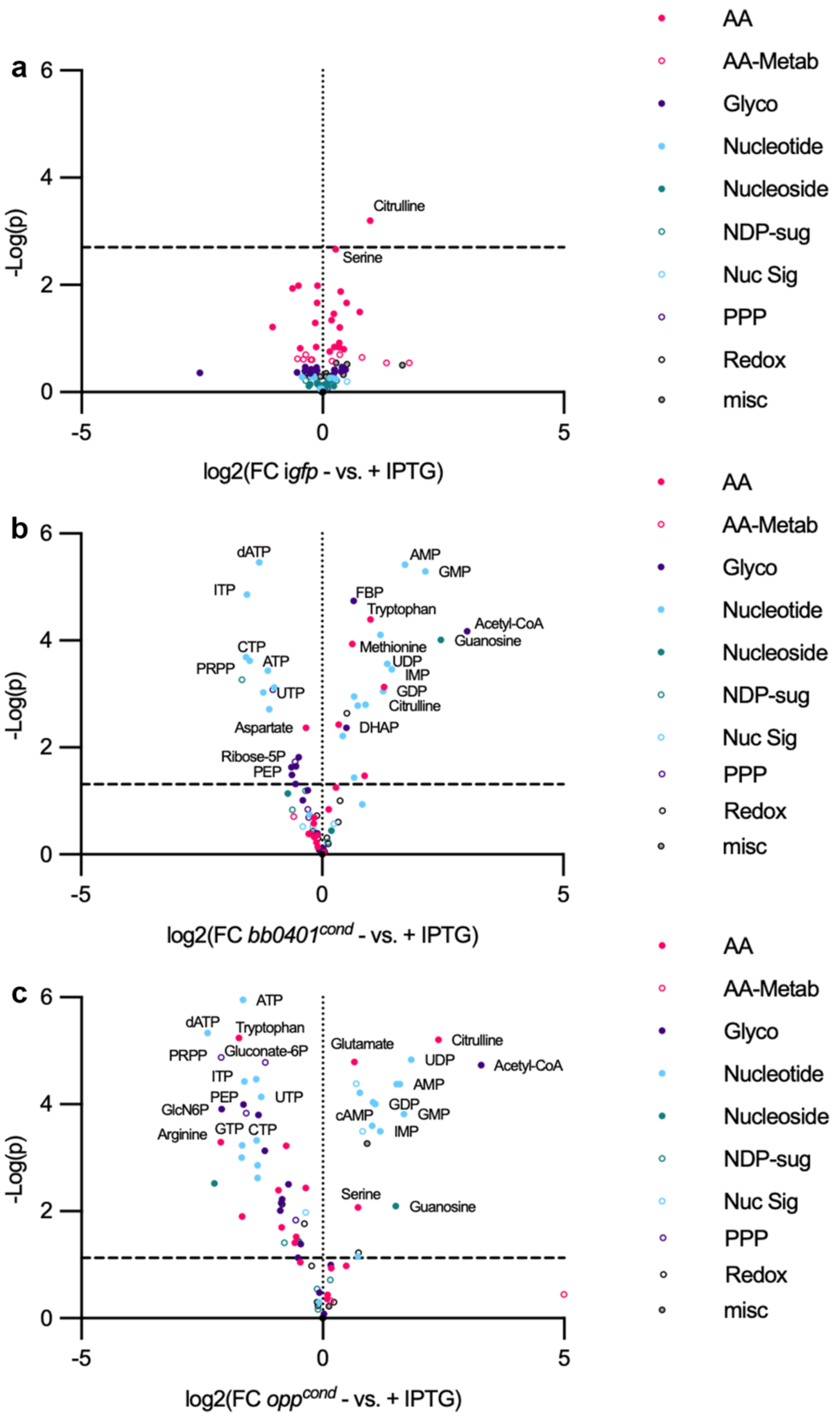

Figure S6: *opp*^{cond} starvation results is a larger metabolite shift than *bb0401*^{cond}. Volcano plots showing metabolite changes in a) i*gfp* control, b) *bb0401*^{cond}, and c) *opp*^{cond} when growth without and with 1 mM IPTG. Metabolites are color-coded by primary pathway. Dotted line represents 10% FDR. Tabulated results can be found in Table S3. AA=amino acids, AA-Metab=amino acid metabolites, Glyco=glycolysis and other carbohydrate, NDP-sug=nucleotide diphosphate sugar conjugates, Nuc Sig=signaling nucelotides, PPP=pentose phosphate pathway, Redox=redox cofactors.

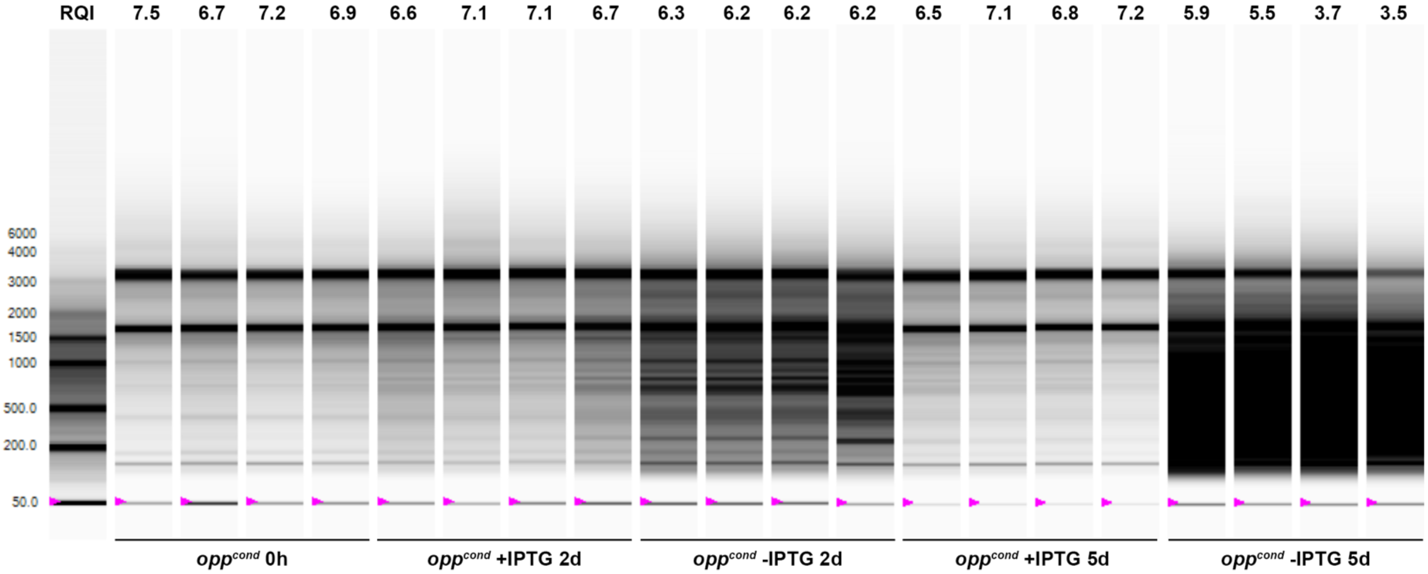

Figure S7: Prolonged starvation of *opp*^{cond} results in RNA degradation. RNA samples collected from *opp*^{cond} at timepoint 0 and without or with 1 mM IPTG at 2 d and 5 d post-incubation with their reported RNA quality indicator (RQI).

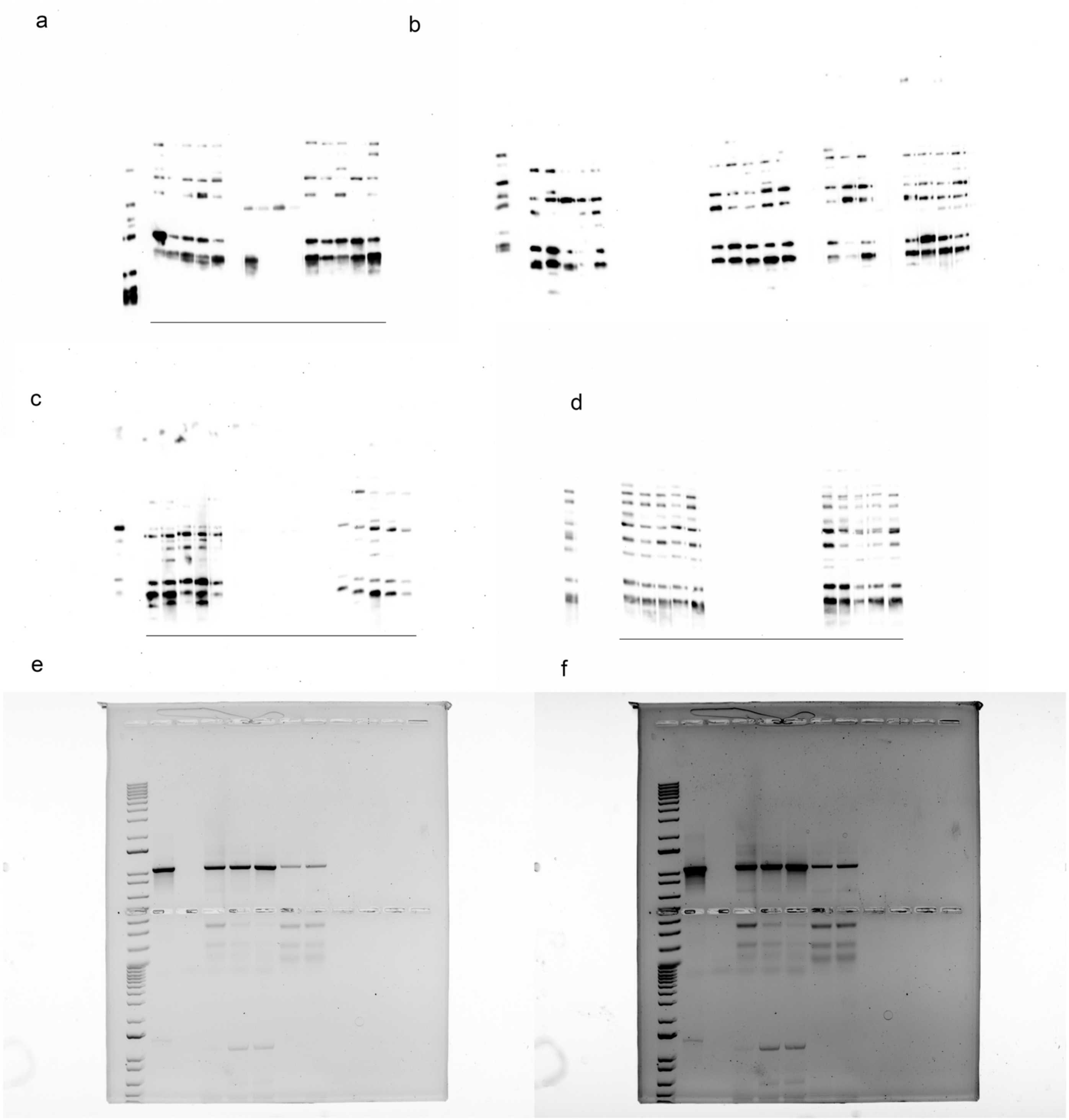

Figure S8: Uncropped gel and blot images. a) Uncropped image of Fig 1a, b) Fig 1b, c) Fig 1g, d) Fig 1h, e) Fig 2c upper panel exposure, f) Fig 2c lower panel exposure.

Supplementary Table 1. *Borrelia burgdorferi* strains and plasmids used in this study

Strain/Plasmid	Description	Antibiotic Resistance	Reference
B. burgdorferi			
BbG100	Wild-type strain B31 5A18 NP1 (*wt*)	Kan	[3]
BbG141A	B31 5A18 NP1 i*bb0334-35* Δ*bb0334-35* (o*pp*^{cond})	Kan/Strep/Erm	[4]
BbG141B	B31 5A18 NP1 i*bb0334-35* Δ*bb0334-35* (o*pp*$^{cond+lp28\text{-}1}$)	Kan/Strep/Erm	This study
BbAG320	B31 5A18 NP1 i*bb0401* (i*bb0401*)	Kan/Strep	This study
BbG119	B31 5A18 NP1 i*bb0401* Δ*bb0401* (*bb0401*^{cond})	Kan/Strep	This study
BbP1781	B31 5A4	N/A	[5]
BbAG367	B31 5A4 i*gfp* (i*gfp*)	Strep	This study
E. coli			
Top10	*F–mcrA Δ(mrr-hsdRMS-mcrBC) φ80lacZΔM15 ΔlacX74 recA1 araD139 Δ(ara-leu)7697 galU galK λ–rpsL(StrR) endA1 nupG*	N/A	Invitrogen
Stellar	*F–, endA1, supE44, thi-1, recA1, relA1, gyrA96, phoA, Φ80d lacZΔ M15, Δ (lacZYA - argF) U169, Δ (mrr - hsdRMS - mcrBC), ΔmcrA, λ–*	N/A	Clonetech
Plasmids			
pUC19	Cloning vector	Amp	Invitrogen
pJSB275	Shuttle vector with IPTG-inducible luciferase lacking NdeI in the resistance marker	Spec/Strep	[4]
pBRV2	Gent marker	Gent	[6]
pEcAG286	pi*bb0401*	Strep	This study
pEcAG259	pΔ*bb0401*	Gent/Amp	This study
pCE320	*gfp*	Zeo	[7]
EcAG304	pi*gfp*	Strep	This study
pG260A	pCR2.1-*mus musculus beta-actin* standard	Kan/Amp	This study
pG148A	pCR2.1-*flaB* standard	Kan/Amp	This study
pG269A	pCR2.1-*bb401* standard	Kan/Amp	This study

Supplementary Table 2. Oligonucleotide primers used in this study

Designation	Sequence (5'-3')	Purpose	Reference
M13 F	CAGGAAACAGCTATGAC	Sequencing	Invitrogen
M13 R	GTAAAACGACGGCCAG	Sequencing	Invitrogen
pless Strep F	ATGAGGGAAGCGGTGATCGCCGA	Diagnostic PCR	6
pless Strep R	TTATTTGCCGACTACCTTGGTG	Diagnostic PCR	6
pless Gent F	ATGTTACGCAGCAGCAACGATG	Diagnostic PCR	6
pless Gent R	TTAGGTGGCGGTACTTGGGTCCA	Diagnostic PCR	6
5' pJSB275 seq	GATTCAATTGTGAGCGGAATAACA	Sequencing	8
3' pJSB275 seq	ATGCGCTTAACGGTAAAATCCAAGG	Sequencing	8
5' bb0401 ind	**GGAGAAATTA*CATATG***AATATAAAAATCAATTTTTTTTTCACTTTG	i*bb0401*cloning	This study
3' bb0401 ind	**CTCTATCTTC*AAGCTT***TTAATTAATTTTTTCTTGATCTTTTAATTCTTTG	i*bb0401*cloning	This study
5' bb0401 null F1	**CGACTCTAGA*GGATCC***GCCTCTTGGCCCTATC	Δ*bb0401* cloning	This study
3' bb0401 null F1	**TTGAAGCTCGGGTAG**ATGACTTCTCCTTTCAGAGATTTA	Δ*bb0401* cloning	This study
5' bb0401 null gent	**GAAAGGAGAAGTCAT**CTACCCGAGCTTCAAGG	Δ*bb0401* cloning	This study
3' bb0401 null gent	**TTAATTTGTTTAGCT**GGCGGTACTTGGGTC	Δ*bb0401* cloning	This study
5' bb0401 null F2	**GACCCAAGTACCGCC**AGCTAAACAAATTAATAGGATTTGGCA	Δ*bb0401* cloning	This study
3' bb0401 null F2	**CGGTACCCGG*GGATCC***GGCCTTTTTTGCGCAC	Δ*bb0401* cloning	This study
5' iGFP	**AAGAGGAGAAATTA*CAT***_ATG_AGTAAAGGAGAAGAACTTTTC	i*gfp* cloning	This study
3' iGFP	**CTCTATCTTC*AAGCTT***TTATTTGTATAGTTCATCCATGCC	i*gfp* cloning	This study
5' actstd	ACCCACACTGTGCCCATC	qPCR std, RT-PCR	This study
3' actstd	GGATGCCACAGGATTCCAT	qPCR std, RT-PCR	This study
5' flaBstd	ATGATTATCAATCATAATACATCAGCTATTAATGCTTCAAG	qPCR std, RT-PCR	This study
3' flaBstd	TTATCTAAGCAATGACAAAACATATTGGGGAACT	qPCR std, RT-PCR	This study
5' bb0401std	ATGAATATAAAAATCAATTTTTTTTTCACTTTGCCTATTGGAATC	qPCR std, RT-PCR	This study
3' bb0401std	TTAATTAATTTTTTCTTGATCTTTTAATTCTTTGAAGTTTATTATATGAATGA TTGC	qPCR std, RT-PCR	This study
mb-act F	GACGGACTACCTCATGAAGATCCT	qPCR	9
mb-act R	CACGCACGATTACCCTCTCA	qPCR	9
mb-act probe	ACCGAGCGTGGCTACAGCTTCATCA	qPCR	9
flaB F	CTTTTCTCTGGTGAGGGAGCTC	qPCR	10
flaB R	GCTGCCTTCCTGTTGAACACCC	qPCR	10
flaB probe	CTTGAACCGGTGCAGCCTGAGCA	qPCR	10
bb0401 F	GCAGAATAGCTAGAGAACTGATGCT	qPCR	This study
bb0401 R	TGTTTACAATAAACCCATTTGCATGGTAAAA	qPCR	This study
bb0401 probe	FAM-AAGCGCATCCAATCTT-NFQ	qPCR	This study

Bold denotes overlap sequence for InFusion cloning. Italics denotes restriction sites.

Supplementary Table 3. Proteins used in phylogenetic tree

Designation	Orgamism	UniProt ID
BaGltP	*Borrelia afzelii*	Q0SNA6
BaTcyP	*Borrelia afzelii*	Q0SMC7
BB0401	*Borrelia burgdorferi*	O51362
BB0729	*Borrelia burgdorferi*	O51671
BdGltP	*Borrelia duttonii*	B5RLW1
BdTcyP	*Borrelia duttonii*	B5RMS2
BgGltP	*Borrelia garinii*	Q661L7
BgTcyP	*Borrelia garinii*	Q660E4
BhGltP	*Borrelia hermsii*	B2S0A5
BhTcyP	*Borrelia hermsii*	B2S171
BmGltP	*Borrelia miyamotoi*	AGT27383
BmTcyP	*Borrelia miyamotoi*	A0AAX3JKT2
BrGltP	*Borrelia recurrentis*	B5RRK9
BrTcyP	*Borrelia recurrentis*	B5RQ68
BstGltT	*Bacillus stearothermophilus*	P24943
BstTcyP	*Bacillus stearothermophilus*	A0A0K9HFS9
BsuGltP	*Bacillus subtilis*	P39817
BsuTcyP	*Bacillus subtilis*	P54596
BtGltP	*Borrelia turicatae*	A1QZI9
BtTcyP	*Borrelia turicatae*	A1R0F3
EcGltP	*Esherichia coli*	P21345
EcGltS	*Esherichia coli*	P0AER8
EcTcyP	*Esherichia coli*	P77529
KpGltP1	*Klebsiella pneumoniae*	A0A377TLH4
KpGltP2	*Klebsiella pneumoniae*	A0A377TW36
KpTcyP	*Klebsiella pneumoniae*	A0ABD7NSB9
PhGltPh	*Pyrococcus horikoshii*	O59010
SaGltS	*Staphylococcus aureus*	X5E058
SaTcyP	*Staphylococcus aureus*	X5E2G5
SLC1A1	*Homo sapiens*	P43005
SLC1A2	*Homo sapiens*	P43004
SLC1A3	*Homo sapiens*	P43003
SLC1A4	*Homo sapiens*	P43007
SLC1A5	*Homo sapiens*	Q15758
SLC1A6	*Homo sapiens*	P48664
SLC1A7	*Homo sapiens*	O00341
SLC7A11	*Homo sapiens*	Q9UPY5
TdTcyP	*Treponema denticola*	Q73RF9
TkGltTk	*Thermococcus kodakarensis*	Q5JID0
TpGltP	*Treponema pallidum*	WGV79979
YpGltP	*Yersinia pestis*	A0AAX2I5T7
YpTcyP	*Yersinia pestis*	A0AAX2I6C1

References

1 Boudker, O., Ryan, R. M., Yernool, D., Shimamoto, K. & Gouaux, E. Coupling substrate and ion binding to extracellular gate of a sodium-dependent aspartate transporter. *Nature* **445**, 387-393 (2007). https://doi.org/10.1038/nature05455

2 Rahman, M. *et al.* Characterisation of the DAACS Family Escherichia coli Glutamate/Aspartate-Proton Symporter GltP Using Computational, Chemical, Biochemical and Biophysical Methods. *J Membr Biol* **250**, 145-162 (2017). https://doi.org/10.1007/s00232-016-9942-x

3 Kawabata, H., Norris, S. J. & Watanabe, H. BBE02 disruption mutants of Borrelia burgdorferi B31 have a highly transformable, infectious phenotype. *Infect Immun* **72**, 7147-7154 (2004). https://doi.org/10.1128/IAI.72.12.7147-7154.2004

4 Groshong, A. M., Dey, A., Bezsonova, I., Caimano, M. J. & Radolf, J. D. Peptide Uptake Is Essential for *Borrelia burgdorferi* Viability and Involves Structural and Regulatory Complexity of its Oligopeptide Transporter. *mBio* **8** (2017). https://doi.org/10.1128/mBio.02047-17

5 Purser, J. E. & Norris, S. J. Correlation between plasmid content and infectivity in Borrelia burgdorferi. *Proc Natl Acad Sci U S A* **97**, 13865-13870 (2000). https://doi.org/10.1073/pnas.97.25.13865

6 Caimano, M. J. *et al.* The RpoS Gatekeeper in *Borrelia burgdorferi*: An Invariant Regulatory Scheme That Promotes Spirochete Persistence in Reservoir Hosts and Niche Diversity. *Front Microbiol* **10**, 1923 (2019). https://doi.org/10.3389/fmicb.2019.01923

7 Eggers, C. H. *et al.* Identification of loci critical for replication and compatibility of a Borrelia burgdorferi cp32 plasmid and use of a cp32-based shuttle vector for the expression of fluorescent reporters in the lyme disease spirochaete. *Mol Microbiol* **43**, 281-295 (2002). https://doi.org/10.1046/j.1365-2958.2002.02758.x

8 Groshong, A. M., McLain, M. A. & Radolf, J. D. Host-specific functional compartmentalization within the oligopeptide transporter during the *Borrelia burgdorferi* enzootic cycle. *PLoS Pathog* **17**, e1009180 (2021). https://doi.org/10.1371/journal.ppat.1009180

9 Groshong, A. M. *et al.* BB0238, a presumed tetratricopeptide repeat-containing protein, is required during *Borrelia burgdorferi* mammalian infection. *Infect Immun* **82**, 4292-4306 (2014). https://doi.org/10.1128/IAI.01977-14

10 Pal, U. *et al.* TROSPA, an Ixodes scapularis receptor for Borrelia burgdorferi. *Cell* **119**, 457-468 (2004). https://doi.org/10.1016/j.cell.2004.10.027
