# Supplementary material for: Dissection of amino acid acquisition pathways demonstrates that amino acid starvation of Borrelia burgdorferi results in a (p)ppGpp-independent maladaptive response
Source: Commun Biol. 2025 Dec 23;9:105. doi: 10.1038/s42003-025-09374-0 (PMC12830385; doi:10.1038/s42003-025-09374-0)
Supplement: Supplementary file 3 — Description of Additional Supplementary Files [file 42003_2025_9374_MOESM3_ESM.pdf]

Description of Additional Supplementary File

File name: Supplementary data 1
Description: Metabolomics

File name: Supplementary data 2
Description: RNAseq

File name: Supplementary data 3
Description: p values

File name: Supplementary data 4
Description: Numerical source data
